# Regional specialization and market integration: agroecosystem energy transitions in Upper Austria

**DOI:** 10.1007/s10113-017-1145-1

**Published:** 2017-04-07

**Authors:** Simone Gingrich, Michaela Clarissa Theurl, Karlheinz Erb, Fridolin Krausmann

**Affiliations:** 1 Institute of Social Ecology Vienna, Universitaet Klagenfurt, Schottenfeldgasse 29, 1070 Vienna, Austria; 2 FiBL Austria, Research Institute of Organic Agriculture, Doblhoffgasse 7/10, 1010 Vienna, Austria

**Keywords:** Agroecosystem energy transition, Agroecosystem energy flows, Long-term socio-ecological research, Energy efficiency, EROI

## Abstract

We investigate agroecosystem energy flows in two Upper Austrian regions, the lowland region Sankt Florian and the prealpine region Grünburg, at five time points between 1830 and 2000. Energetic agroecosystem productivity (energy contents of crops, livestock products, and wood per unit area) is compared to different types of energy inputs, i.e., external inputs from society (labor, industrial inputs, and external biomass inputs) and biomass reused from the local agroecosystem (feed, litter, and seeds). Energy transfers between different compartments of the agroecosystem (agricultural land, forest, and livestock) are also quantified. This allows for delineating an agroecosystem energy transition: In the first stage of this transition, i.e., in the nineteenth century, agroecosystem productivity was low (final produce ranged between 14 and 27 GJ/ha/yr), and local biomass reused made up 97% of total energy inputs in both regions (25–61 GJ/ha/yr). In this period, agroecosystem productivity increase was achieved primarily through more recycling of energy flows within the agroecosystems. In the second stage of the agroecosystem energy transition, i.e., after World War II, external energy inputs increased by factors 2.5 (Sankt Florian) and 5.0 (Grünburg), partly replacing local energy transfers. Final produce per area increased by factors 6.1 (Sankt Florian) and 2.9 (Grünburg). The difference in the resulting energy returns on investment (EROI) owes to regional specialization on cropping versus livestock rearing and to increasing market integration. Our results suggest that sustainable land-use intensification may benefit from some regional specialization harnessing local production potentials based on a mix of local and external inputs.

**Electronic supplementary material:**

The online version of this article (doi:10.1007/s10113-017-1145-1) contains supplementary material, which is available to authorized users.

## Introduction

The past centuries have been characterized by fundamental changes in global land use which went along with population growth and changes in biomass demand. Agricultural land, i.e., the sum of cropland and pasture land increased from 4% of the global ice-free surface in 1700 to 35% in 2000 (Klein Goldewijk et al. 2011). In addition, land-use intensification has changed the way in which agricultural and forest land was used. Between 1900 and 2000, global biomass extraction increased 3.6-fold (Krausmann et al. 2009), and global aboveground Human Appropriation of Net Primary Productivity (HANPP) doubled from 13% of potential net primary productivity in 1910 to 25% in 2005 (Krausmann et al. 2013).

Energy played a crucial role in these land-use changes, an interrelation conceptualized in the “socio-ecological transition” (Fischer-Kowalski and Haberl 2007). Evidence from various long-term case studies suggests that land-use intensification increased land productivity, i.e., the amount of energy harvested per unit of area, at the expense of declining labor productivity, i.e., the amount of energy harvested per unit of labor energy input (Boserup 1965; Ringhofer et al. 2014). From the early and mid-twentieth century onward, fossil-fuel-based technology introduced a new external energy source to agriculture and forestry, and the link between land and labor productivity was weakened. Energy inputs into agriculture by ways of fossil-fuel-based machinery, mineral fertilizers, and other industrial inputs grew rapidly and reached levels similar to those of crop harvests in the year 2000 at the global scale (Smil 2000). Industrial energy inputs also contributed to growing ecological impacts of land use, including soil degradation, ground water pollution, and biodiversity loss (Millennium Ecosystem Assessment 2005). Several national case studies have shown that energy inputs into agroecosystems have by and large matched increases in energetic outputs in recent decades, resulting in stable (Canada), slightly declining (Turkey, Spain), or slightly increasing (USA) energy returns on investment (Guzman Casado et al. 2017; Ozkan et al. 2004; Hamilton et al. 2013).

This study contributes to a recent endeavor at understanding regional-scale long-term trajectories of agroecosystem energetic efficiency in the course of socio-ecological transitions (Tello et al. 2016; Galan et al. 2016). We use empirical evidence from two Central European case studies at five points in time between 1830 and 2000 to trace temporal trends of intensification and industrialization on agroecosystem energetics. We address the question to which degree increases in agroecosystem productivity were achieved at the expense of increasing energy flows within the agroecosystem or external, fossil or biotic, energetic inputs. The long-term comparison of two regions which are located closely, but differ in terms of topography and soil, contributes new insights into temporal trajectories and spatial divergences of agroecosystem energy transitions. Our results are discussed in view of sustainable land-use intensification.

## Methods

### Case studies and concept

The two regions investigated represent two different biogeographical regions of Central Europe, located in the Austrian province Upper Austria and only c. 30 km apart, but in different agricultural production zones (Wagner 1990). Sankt Florian is situated in the “Alpenvorland,” a productive area south of the Danube river characterized by lowlands on fertile soils. The other region, Grünburg, is located in the “Voralpen,” the hilly northern fringes of the Alps, along a gradient from lowland to mountainous, with steeper slopes and less favorable soils (SI Fig. 1). The case studies offer great potential for comparative analyses because they were managed under similar legal and institutional conditions throughout the period, but differ in terms of their biogeographic potentials. From pre-industrial mixed farming with differing yields, the regions specialized on high-yielding cropping and pig and poultry rearing (Sankt Florian), and on a mix of cropping and grassland-based cattle rearing with a higher share of organic farms (Grünburg, see Kirner et al. 2002).

Following the methodology developed in Tello et al. (2015, 2016), we reconstruct a set of indicators of agroecosystem energetics. The analysis is rooted in long-term socio-ecological research (Haberl et al. 2006; Singh et al. 2013) and adopts a socio-metabolic perspective (Gonzalez de Molina and Toledo 2014), investigating the energetic exchange between rural communities and their agroecological environment. We thus focus on biophysical indicators, i.e., energy input or output per unit of agroecological area and energy output per unit of energy input. Disaggregating different groups of energy input and output according to their type and origin enables us to portray different aspects of energetic efficiency of land-use intensification under differing agroecological conditions and during different stages of industrialization. A monetary analysis (of, e.g., labor productivity or total factor productivity) is beyond the scope of this study.

**Fig. 1:**
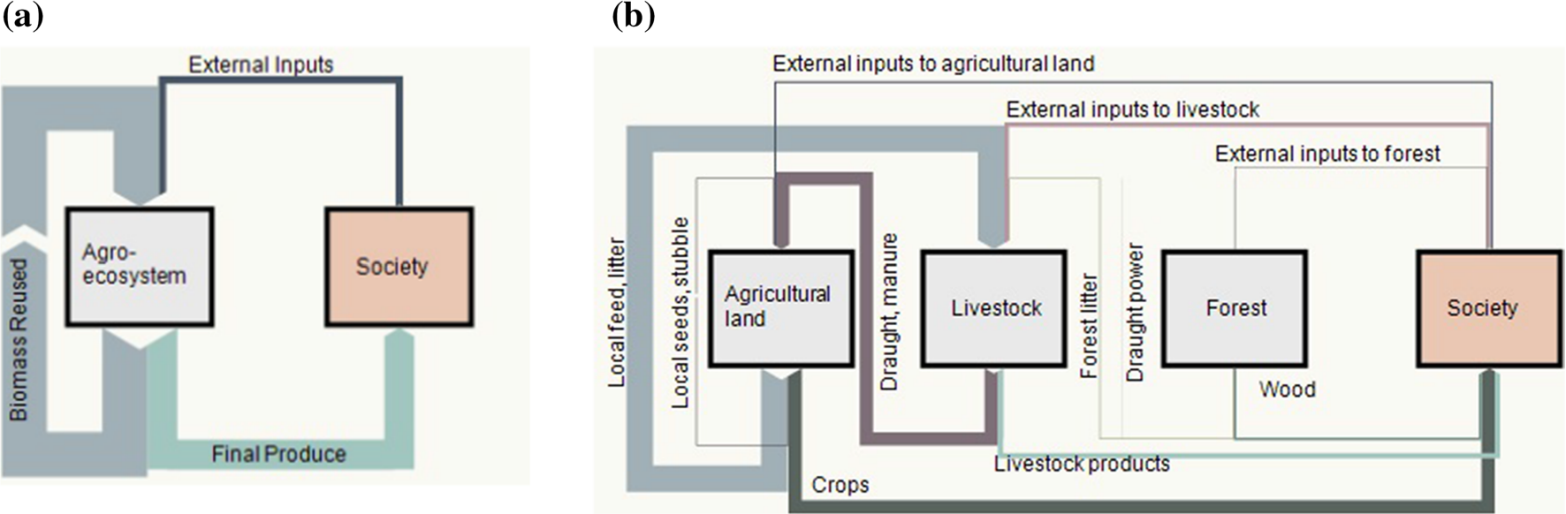
Energy flows considered in this study. **a** simplified model of energy flows between agroecosystem and society according to Tello et al. (2016). **b** breakdown of “agroecosystem” into agricultural land, livestock, and forest, allowing to depict flows between these compartments

The empirical analysis aims at depicting socio-ecological energy flows into, out of, and within the agroecosystem, with the exception of solar radiation (Fig. 1a). This includes (1) the energy content of agricultural products produced in the region and consumed by local farmers or sold on the market (“final produce”, FP), comprising crops, livestock products and wood, or agricultural residues if they are sold outside the region, and (2) direct and indirect socioeconomic energy inputs into the agroecosystem used to generate FP. Acknowledging that large fractions of biomass extracted from the agroecosystem are reinvested into the local agroecosystem (as seeds and stubble, feed and litter), we differentiate two types of energy inputs: (a) external inputs (EI), including labor, industrial inputs, and biomass stemming from outside the regional agroecosystem, and (b) biomass reused (BR), comprised of locally used seeds, feed, grazed biomass and litter, as well as stubble ploughed into soils. Unused biomass is not accounted for in this study.

Based on these energy flow data, we account for three interrelated energy returns on investment (EROI), dividing FP by different types of energetic inputs, i.e., (1) External Final EROI (EFEROI); (2) Internal Final EROI (IFEROI); and Final EROI (FEROI). The three indicators are defined as follows:

1. EFEROI = FP/EI.
2. IFEROI = FP/BR.
3. FEROI = FP/(EI + BR).

EFEROI is similar to many traditional EROI indicators, defined as the ratio of energy generated (in our case: final produce, FP) to the amount of external energy inputs required to generate this energy, in our case external inputs, EI (Hall et al. 2009). IFEROI in contrast is the ratio of FP to biomass reused the agroecosystem’s internal energy flows which are purposefully recycled by society (BR). FEROI is the ratio of FP to total inputs, including both EI and BR. The three EROIs allow to trace not only changes in overall energy efficiency of agroecosystem production, but also consider the functional and qualitative differences of energy inputs, as well as scale shifts from local recycling to external inputs. For a detailed definition of concepts and terminology of the three EROIs, see Tello et al. (2016, 2015) and Galan et al. (2016).

In a more detailed investigation, we additionally discern energy flows according to their origin and destination, distinguishing “society” and three functionally different compartments within the agroecosystem: agricultural land (including grassland and cropland), forest, and livestock (Fig. 1b). By considering livestock as part of the agroecosystem, rather than as part of “society,” our system boundary differs from the one commonly employed in socio-metabolic material or energy flow accounting (Fischer-Kowalski et al. 2011).

### Data and accounting procedures

Different source types provide the core data of our analysis: For 1830, the main source is the Franciscean, or Franciscan, Cadastre (Sandgruber 1979), an archival source presenting village-level data on land use, yields, seed, and manure output and livestock numbers (AT-OOeLA 1830). The names of the villages and their cadastral source details are provided in the supplementary information (SI Table 1). For the period from 1864 to 2000, a previous study by the authors quantifies energy flows in agricultural production, as well as direct energy inputs (Gingrich et al. 2013). This study relies on Lorenz (1866) as major source for the year 1864, and village-level agricultural statistics by Statistik Austria and its precursory organization, accessible in individual publications or the online database (ISIS database). Data from this previous work were revised, reallocated, and extended to account for the EROI indicators presented here. The names of the villages are provided in the supplementary information (SI Table 2).

Tables 1, 2, and 3 present the data sources used, the processing steps, and underlying assumptions to arrive at agroecosystem energy flow values and establish EROI indicators. Outputs from the agroecosystem are a well-documented energy flow (Table 1). We establish the production of primary crops based on land-use data, crop, and wood yields. Crop residues are a large energy flow, which was mostly used locally as feed or litter (biomass reuse). In the late-twentieth century, crop residues were partly exported from the region and were then accounted as final produce. We estimate the amount of exported residues in a demand-based approach, assuming that all residues which were neither used by livestock (see below) nor ploughed into the soil were sold on markets, and corroborated this assumption by the literature (Dissemond and Zaussinger 1995). Livestock final production is estimated based on livestock numbers, live weight, slaughter rates, and, if available, actual livestock production data.

**Table 1:** Data sources used and data processing steps performed: Final produce from the agroecosystem (FP)

Energy flow	Data	Year of reference	Source	Assumptions and processing steps
Crop and wood production	land use, yields	1830	Franciscean Cadastre	Conversion of fresh weight into energy (Haberl 1995)
		1864	Lorenz 1866	
		1949–2000	Österreichisches statistisches Zentralamt 1950; ISIS database	
Livestock production for society	Livestock numbers, live weight, slaughter rate, animal production	1830	Franciscean Cadastre, Hitschmann 1891	Conversion of livestock products into energy (Krausmann 2008)
		1864	Lorenz 1866	
		1950	Österreichisches statistisches Zentralamt 1952a	Conversion of livestock products into energy (Festersen 1990; Darge 2001)
		1960–2000	ISIS database

**Table 2:** Data sources used and data processing steps performed: external inputs (EI) into the agroecosystem

Energy flow	Data	Year of reference	Source	Assumptions and processing steps
Labor	Land use,	1830	Franciscean cadastre	Area and species-specific information on labor time requirements (Hitschmann 1891), gross calorific value of energy in food intake per hour (Darge 2001)
	livestock numbers	1864	Lorenz 1866	
	agricultural population	1951	Österreichisches Statistisches Zentralamt 1952b	Annual work time per agricultural worker, gross calorific value of energy in food intake per hour (Darge 2001). Share of non-local food consumption (own estimate: 1950 and 1960: 70%, 2000: 99%) and energy required in transport and processing (Steinhart and Steinhart 1974)
		1949–2000	ISIS database	
Other external inputs to agricultural land and forest	Kitchen waste	1830–1864	Local food consumption	5% of vegetable production was assumed to be used as compost.
		1950–2000	Agricultural population	5% of the agricultural population’s food demand was assumed to be used as compost
	Fossil fuels	1950–2000	(Darge 2001)	Total Austrian fossil fuel use in agriculture and forestry allocated to regions based on per-area values; energy embodied in fossil fuels (Aguilera et al. 2015)
	Fertilizer	1950–2000	Estimate based on national Austrian fertilizer use (Austrian Institute of Economic Research database) and crop-specific fertilizing recommendations (Löhr 1952; Ruhr-Stickstoff-Aktiengesellschaft 1957; BMLF 1999)	Conversion of fertilizer output into (embodied) energy flows (Aguilera et al. 2015)
	Pesticides	1950–2000	FAOstat (2000), own estimates based on 2000 value: 1960: 50% of 2000; 1950: 50% of 1960	Total Austrian pesticide use allocated to regions. Conversion of pesticides output into (embodied) energy flows (Aguilera et al. 2015)
	Market seeds	1950–2000	Based on per-area demand of seed output (Löhr 1983)	A fraction of seed demand was assumed to be derived from non-regional markets (1950: 20%, 1960: 50%, 2000: 100%). Energy embodied in market seeds was roughly estimated as 5% of energy content in 1960 and 8% in 2000
Other external inputs to livestock	Electricity	1950–2000	(Darge 2001)	Total Austrian electricity use in agriculture allocated to regions based on per-ha values. Embodied energy in electricity generation was assessed by applying technology-specific data provided by Aguilera et al. (2015) to the Austrian electricity mix provided in Staitistik Austria’s online database
	Market feed	1950–2000	Based on feed demand (Löhr 1952)	Market feed was assessed as difference between supply and demand (1950, 1960), and as 33% of demand in 2000 (Gingrich et al. 2013). Energy embodied in market feed was roughly estimated as 5% of energy content in 1960 and 8% in 2000
	Market litter	2000	Based on litter demand (BMLFUW 2006)	In Grünburg in 2000, litter demand substantially exceeded straw production, and market litter was assumed as the difference between demand and local supply

**Table 3:** Data sources used and data processing steps performed: Biomass reuse and agroecosystem energy transfers

Energy flow	Data	Year of reference	Source	Assumptions and processing steps
Local feed reuse	Livestock numbers, species-specific feed demand	1830	Franciscean Cadastre; Hitschmann 1891	Local feed reuse comprises locally produced fodder, straw not used as litter, hay, and grazed biomass. Fodder and hay harvest was derived from sources (see crop and wood production). Grazed biomass was assumed to amount to the difference between local demand and supply (1830–1864). In 2000, local feed was assumed to account for only 66% of demand. Conversion to energy (Haberl 1995)
		1864	Lorenz 1866	
		1950	Österreichisches statistisches Zentralamt 1952a; Löhr 1952	
		1960–2000	ISIS database; Löhr 1952	
Local litter reuse	Livestock numbers, species-specific litter demand for straw	1830	Franciscean Cadastre; Lorenz 1866	Straw production was assessed based on grain production and harvest indices (1830–1864) and derived from statistical records in later years (Österreichisches statistisches Zentralamt, 1950; ISIS database). Litter was assumed to be of local origin unless demand exceeded production; conversion to energy (Haberl 1995)
		1864	Lorenz 1866	
		1950	Österreichisches statistisches Zentralamt 1952a; BMLFUW 2006	
		1960–2000	ISIS database; BMLFUW 2006	
Forest litter reuse	Livestock numbers, species-specific litter demand for straw	1830	Franciscean Cadastre; Lorenz 1866	Lorenz (1866) provides region- and species-specific information on forest litter use. In Grünburg in 1950, 50% of the difference between litter demand and local production was assumed to be covered by forest litter
		1864	Lorenz 1866	
		1950	Österreichisches statistisches Zentralamt 1952a; BMLFUW 2006	
Local seeds	Land use data, crop-specific seed demand	1830	Franciscean Cadastre	In the Franciscean Cadastre and Lorenz (1866), seed output is stated and was assumed to be of local origin entirely
		1864	Lorenz 1866	
		1950	Österreichisches statistisches Zentralamt 1952a; Löhr 1983	A fraction of seed demand was assumed to be derived from local sources (1950: 80%, 1960: 50%). In 2000, all seeds were assumed to be from outside sources
		1960-2000	ISIS database; Löhr 1983	
Local stubble	Land area used for grain production	1830–2000	See local litter reuse	10% of straw production was assumed to be ploughed into the soil
Manure consumption	Livestock numbers, feed intake	1830	Franciscean Cadastre; Hitschmann 1891	In 1830 and 1864, manure was assessed based on feed intake, considering the amount of time spent in stables (Hitschmann 1891). Manure was converted into energy (Darge 2001). All manure was assumed to be applied locally
		1864	Lorenz 1866; Hitschmann 1891	
	Livestock numbers, species-specific manure production	1950	Österreichisches statistisches Zentralamt 1952a; BMLFUW 2006	In 1950–2000, species- and age-specific manure production values were applied and converted into energy (Darge 2001). All manure was assumed to be applied locally
		1960–2000	ISIS database; BMLFUW 2006	
Draught power	Land use data; land-use specific draught demand	1830	Franciscean Cadastre; Hitschmann 1891	Draught power requirements for different land-use types were derived from Hitschmann (1891) and applied to the regions, and cross-checked with livestock numbers and work capacities. The energy content of draught power was defined as the share of feed energy necessary to supply for the time spent on draught
		1864	Lorenz 1866; Hitschmann 1891	
	Number of draught animals	1950	Österreichisches statistisches Zentralamt 1952a	The number of draught animals was multiplied with their power and estimates of annual work time per draught animal (500 h/yr for draught oxen and bulls, 600 h/yr for draught horses, and 300 h/yr for draught cows)
		1960	ISIS database; BMLFUW 2006	

The primary data used to calculate external energy inputs into the agroecosystem (Table 2) are the least robust used in the calculation. No regional or sectoral data directly reporting agroecosystem energy inputs are available. Therefore, we estimate energy flows based on the associated agroecosystem structure (land use, livestock, machinery, agricultural work force). In the nineteenth century, we account only for labor and kitchen waste as the only external energy input, neglecting the minor quantities of energy embodied in iron-based agricultural tools. We base our labor estimates on the most robust data available. In the nineteenth century, this is information on land use, livestock and estimates of typical labor demand for land and livestock related activities. In the twentieth century, we use the number of agricultural workers and their typical annual work time. Labor is accounted as gross food intake per hour worked (Fluck 1992). In the twentieth century, when food stems increasingly from outside the region, we also consider energy embodied in food production in the form of transport, packaging, and cooling.

In the twentieth century, external energy inputs into the agroecosystem increased in amount and variety. We consider direct and indirect energy inputs in the form of fossil fuels, mineral fertilizer, pesticides, electricity, and biomass in purchased seeds, feed and litter, plus kitchen wastes. In our estimates of industrial energy inputs (fossil fuels, mineral fertilizer, pesticides, electricity), we combine the best available information. This implies downscaling Austrian national data to the agricultural land in the region by applying national averages (e.g., fuel use in tractors per hectare agricultural land). If possible, we complement top-down approaches by bottom-up estimates to grasp potential differences between the two regions. For fertilizers, for example, we build an estimate based on national averages of fertilizer use and another based on crop-specific fertilizing recommendations and derive final values from these two data points for each region and year.

The biomass fraction of external energy inputs is assessed based on local supply and demand balances, assuming that the fraction of local demand of feed and litter which could not be met by local supply was imported from other regions (i.e., accounted for as external input). The agroecosystem’s demand for biomass is arguably a relatively robust estimate, but the share of local versus external consumption had to be estimated roughly, based on expert interviews (see Gingrich et al. 2013). We consider the shares of the different flows to be represented rather solidly, but the actual regional differences not owing to agricultural structure, but to individual farmers’ decisions, are not depicted in our study.

Finally, we account for energy flows within the agroecosystem, including local feed and litter consumption from cropland and forests, grazed biomass, stubble ploughed back into soils, manure output and draught power use on agricultural land and forests (Table 3). We consider the assessments of these flows as rather robust, given that they rely mostly on regional data (e.g., data on actual feed and litter demand in 1864 and data on the number of draught animals in 1950 and 1960) or well-established accounting procedures (e.g., feed balances based on livestock numbers and livestock weight).

The use of such a diversity of sources entails two specific consistency problems. (1) Data caveats in the nineteenth century need to be considered. As a fiscal source, the Franciscean cadaster (AT-OOeLA 1830) may underestimate actual land productivity, or even livestock numbers (Granda 2006). Lorenz (1866), on the other hand, using manorial records for estimating regional production, may overestimate production. This may result in an overestimation of growth in output and productivity improvements in the nineteenth century. (2) Data in the twentieth century refer to a larger region than in the nineteenth century: In twentieth century censuses, data are no longer available at the village scale, but at the level of political communities, or at even higher scales, such as judicial or political districts. In order to derive comparable data, we chose political communities of similar topology as the original regions. The size of the case study regions in the twentieth century exceeds the nineteenth century size by up to 65% (Sankt Florian) and 93% (Grünburg). In addition, the principle of land-use data assessment changed between the nineteenth and twentieth centuries: While our nineteenth century sources report land area actually located within the respective village boundaries, the agricultural census of the twentieth century presents land area managed by local farmers, rather than total land area within the political boundaries. This entails changes in total land area covered in our data set (the result of expansion or contraction of individual farms). We address these problems by comparing only relative numbers over time, i.e., agroecosystem productivity in GJ/ha/yr or energetic efficiency in GJ/GJ. We trust the relative data to be comparable in the long run, given that the agricultural structure of the larger areas was similar to that of the smaller ones in both the early-nineteenth century and recently, as supported by a visual check with cadastral maps and recent areal photographs available online.^1^

## Results

Overall, the agroecosystem structure of the two regions diverged throughout the time period, with most pronounced change taking place in the last decades of the twentieth century (Table 4). Both regions were characterized by mixed farming in the early-nineteenth century, with mosaics of forests, grassland, and cropland. Cropland was managed as three-field rotation system in both regions throughout the nineteenth century, and important cereals included wheat, rye, and oats. Land use in Sankt Florian was more productive, with higher shares of cropland and higher yields throughout the period of observation. In the late 20th century, Sankt Florian focused on intensive cropping, with sugar beet and corn adding to cereal production, which was increasingly dominated by wheat. In Grünburg, we observe a gradual shift from cropland to grassland over time, and the remaining cropland was used for cereal, potato, and corn production in the late-twentieth century. Livestock density was similar in the two regions until the mid-twentieth century. Livestock numbers were dominated by cattle in both regions, with diverging relevance of horses, sheep and pigs. In the late-twentieth century, farmers in Sankt Florian largely abandoned cattle rearing and specialized on pig and chicken production, while farmers in Grünburg increased both cattle and pig rearing, resulting in much higher livestock densities.

**Table 4:** Agricultural structure in Sankt Florian and Grünburg 1830–2000 *Sources* see Tables 1, 2 and 3

	St Florian					Grünburg				
	1830	1864	1950	1960	2000	1830	1864	1950	1960	2000
Area (km^2^)	49	50	80	80	67	59	59	101	99	89
Population density (cap/km^2^)	81	94	182	168	358	90	88	90	84	107
Agricultural population (%)	39	43	13	8	3	30	31	25	18	13
Share of cropland (%)	68	65	57	60	77	40	39	31	31	31
Share of grassland (%)	15	17	25	21	5	32	34	47	45	37
Share of forest (%)	17	18	18	19	18	28	27	22	24	30
Cereal yields (t/ha/yr)	1.2	1.6	1.7	2.6	6.5	0.9	1.6	1.5	2.1	5.5
Livestock density (LSU/km^2^)*	31	55	51	63	30	28	40	51	64	87
Share ruminants (% LSU)*	61	66	66	74	14	91	88	78	84	71

* LSU refers to standardized livestock units of 500 kg live weight

**Fig. 2:**
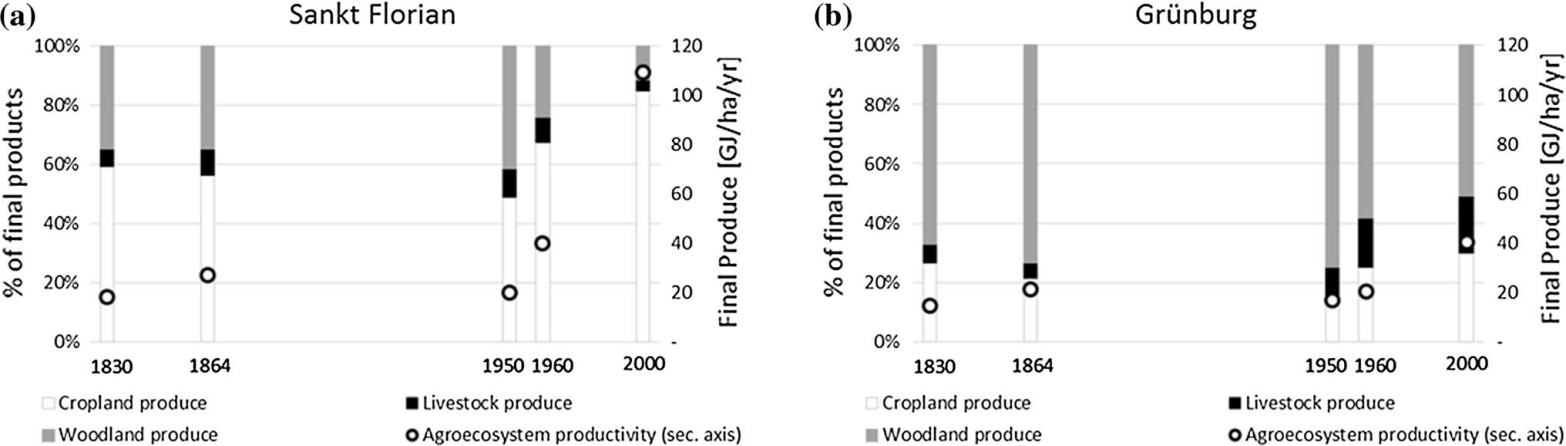
Agroecosystem productivity in St. Florian (**a**) and Grünburg (**b**), 1830–2000: share of different product types in final produce (*left axis*) and agroecosystem productivity (*right axis*)

The amount of energy flowing into and out of the agroecosystems changed during the time period under investigation, and so did the relative importance of the different energy flows. Agroecosystem productivity (i.e., final produce per unit of area in a given year) increased significantly in both regions between 1830 and 2000, from 18 to 109 GJ/ha/yr in Sankt Forian, and from 15 to 40 GJ/ha/yr in Grünburg (Fig. 2). In both regions, agroecosystem productivity increased slightly in the nineteenth century. The slight decline between 1864 and 1950 is related to the effects of post-war cropland abandonment, rather than reflecting an actual long-term trend. In the second half of the twentieth century, a more rapid increase in agroecosystem productivity set in in both regions, though much more pronounced in Sankt Florian.

Two major factors contributed to the different levels of agroecosystem productivity and their change over time: (1) The distribution of agricultural production among cropland, livestock, and woodland products diverged in the course of regional specialization during the late-twentieth century. In the nineteenth century, the share of livestock products was similar in the two regions (5 and 9% of final produce). The higher share of cropland products in Sankt Florian (resulting from both higher cropland shares and higher yields) was compensated by higher forest shares in Grünburg which provided slightly higher yields than croplands at the time. In the second half of the twentieth century, when livestock products gained relative importance in Grünburg (19% of final produce in 2000), agroecosystem productivity fell behind the levels of Sankt Florian. Agroecosystem productivity in Sankt Florian, increasingly focusing on intensive cropping, reached more than twice Grünburg’s level by 2000. This is linked to the high gains in crop yields, as well as to the continuously low energy conversion efficiency of livestock rearing. Interestingly, cereal yields actually converged in the two regions (Table 4), hinting at an intra-regional concentration of cropping to the most suitable plots, a process resulting from increasing pressure on farmers to raise land productivity during industrialization (Mather and Needle 1998).

The second major explanation for diverging trends in agroecosystem productivity is partly linked to regional specialization and refers to (2) increasing market integration of agricultural production, affecting in particular the role of straw. Straw was used in stables for litter in substantial amounts throughout the time period in both regions until the final benchmark year. In 2000, straw was added to final produce in Sankt Florian, because local demand for litter was below local production, and straw was sold to other regions (Dissemond and Zaussinger 1995). According to our estimates, straw accounted for 22% of final produce in Sankt Florian in 2000. This is the only case in which final produce contains products very likely entering the livestock sector in a different region (if not used, e.g., for energy generation). If we adopted a different allocation scheme, grouping all biomass (likely) entering the livestock sector on the one hand, and biomass used by humans directly on the other, final produce trends and levels would thus be the same, with the exception of Sankt Florian in 2000. In this case, agroecosystem productivity in Sankt Florian would still have been twice as high as in Grünburg.

**Fig. 3:**
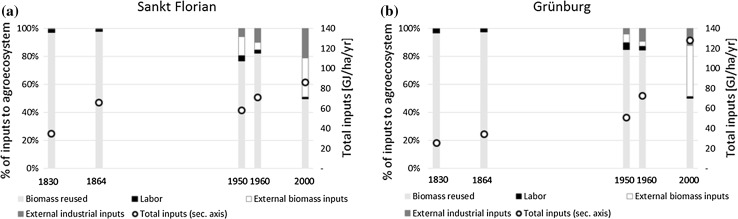
Energy inflows into the regional agroecosystems of Sankt Florian (**a**) and Grünburg (**b**), 1830–2000: share of different types of inflows (*left axis*) and total inflows per area (*right axis*)

External inputs into the agroecosystems of both regions also increased substantially between 1830 and 2000 (Fig. 3). Contrary to outputs, energy inputs grew more in Grünburg (factor 5.0) than in St. Florian (factor 2.5). The total per-area energy inputs into the agroecosystems were in the same order of magnitude as agroecosystem productivity, between 25 and 130 GJ/ha/yr. Throughout the time period, biomass reused, comprising mostly local feed and litter, made up the largest share of energy inputs, the lowest fraction being 49% in Sankt Florian in 2000. Differences between the two regions in the nineteenth century owe to differences in livestock management and cropping, resulting in more straw availability and litter use in stables in Sankt Florian and increasing overall energy input. By 1950, industrial inputs started to be used in agriculture in both regions, subsidized by the international aid programs “United Nations Relief and Rehabilitation Administration” and “European Recovery Program” (Hoffman 1974). In absolute terms, external industrial and biomass inputs were the major drivers for growing energy inputs in both regions in the late-twentieth century. Biomass reused remained stable (Sankt Florian) or increased linearly (Grünburg).

 In 2000, the amount and composition of external inputs differed in the two case studies according to their regional specialization: In Grünburg, where livestock played an important role in the late-twentieth century, imported feed and litter accounted for 46 GJ/ha/yr, which is about one-third of total agroecosystem energy inputs and 74% of external inputs. In Sankt Florian, feed and litter made up for just over one quarter of total inputs, and just over half of external inputs. Instead, inputs of fertilizer and fuels were slightly higher in Sankt Florian in absolute terms at 20 GJ/ha/yr (as opposed to 17 GJ/ha/yr in Grünburg) and much more important in relative terms in 2000. Overall, our results demonstrate that a specialization on industrialized cropping requires less energetic inputs than one on industrial livestock rearing, owing to the high energetic value of feed imports.

When comparing final produce to the different types of inputs, we obtain three distinct but interrelated measures for the energy return on investment (EROI, Table 5). External Final EROI, i.e., the ratio of final produce to external inputs, declined substantially over the 170-year period. In the nineteenth century, External Final EROI ranged between 17 and 25. Between 1830 and 1864, there appears to be an increase in External Final EROI in both regions, which owes largely to higher yields and higher livestock production. Due to the above-described potential biases in the sources, this efficiency increase may be overestimated. Still, the mechanisms of change are depicted: Without a significant increase in external energy input (except labor, which is qualitatively important but not a quantitatively large energy flow, and minor amounts of kitchen wastes), increase in livestock productivity, cropland, or forest yields translates directly into higher External Final EROI in this period. With the introduction of industrial inputs after WWII, External Final EROI dropped dramatically, to 0.6 Grünburg (i.e., external inputs exceeded final produce), and stabilized in Sankt Florian at 2.5. In the crop-specialized region of Sankt Florian, rising external inputs thus matched agroecosystem productivity increase in the late-twentieth century.

**Table 5:** Energy return on investment (EROI) in Sankt Florian and Grünburg 1830–2000

	1830	1864	1950	1960	2000
Sankt Florian					
External final EROI	17.1	19.9	1.5	3.1	2.5
Internal final EROI	0.5	0.4	0.4	0.7	2.5
Final EROI	0.5	0.4	0.3	0.6	1.3
Grünburg					
External final EROI	17.0	25.4	1.9	1.8	0.6
Internal final EROI	0.6	0.7	0.3	0.3	0.6
Final EROI	0.5	0.6	0.3	0.3	0.3

External final EROI is final produce per unit of external inputs to the agroecosystem; Internal final EROI is final produce per biomass reused; Final EROI is final produce per external inputs plus biomass reused. Own calculations, see text

Internal Final EROI, i.e., final produce per unit of biomass reused, remained between 0.3 and 0.7 in both case studies until 1960 and rose to 2.5 in Sankt Florian in 2000, while reaching only 0.6 in Grünburg. This reflects the differences in the importance of livestock production in the two regions. With two-thirds of feed stemming from local production and a high livestock density, Grünburg still retained an important biomass flow within its agroecosystem. The exact amount of feed stemming from outside the regions relies on a number of assumptions and may not be accurately quantified in this study. However, feed demand in both regions significantly exceeded local fodder production in 2000, pointing to a structural dependence on remotely produced fodder. With biomass reused dominating agroecosystem energy inputs, Final EROI, that is final produce per total energy inputs, shows a similar trend as Internal Final EROI until the late-twentieth century, ranging between 0.3 and 0.7. Only in Sankt Florian in 2000 did it exceed 1, i.e., final produce was greater than total inputs.

**Fig. 4:**
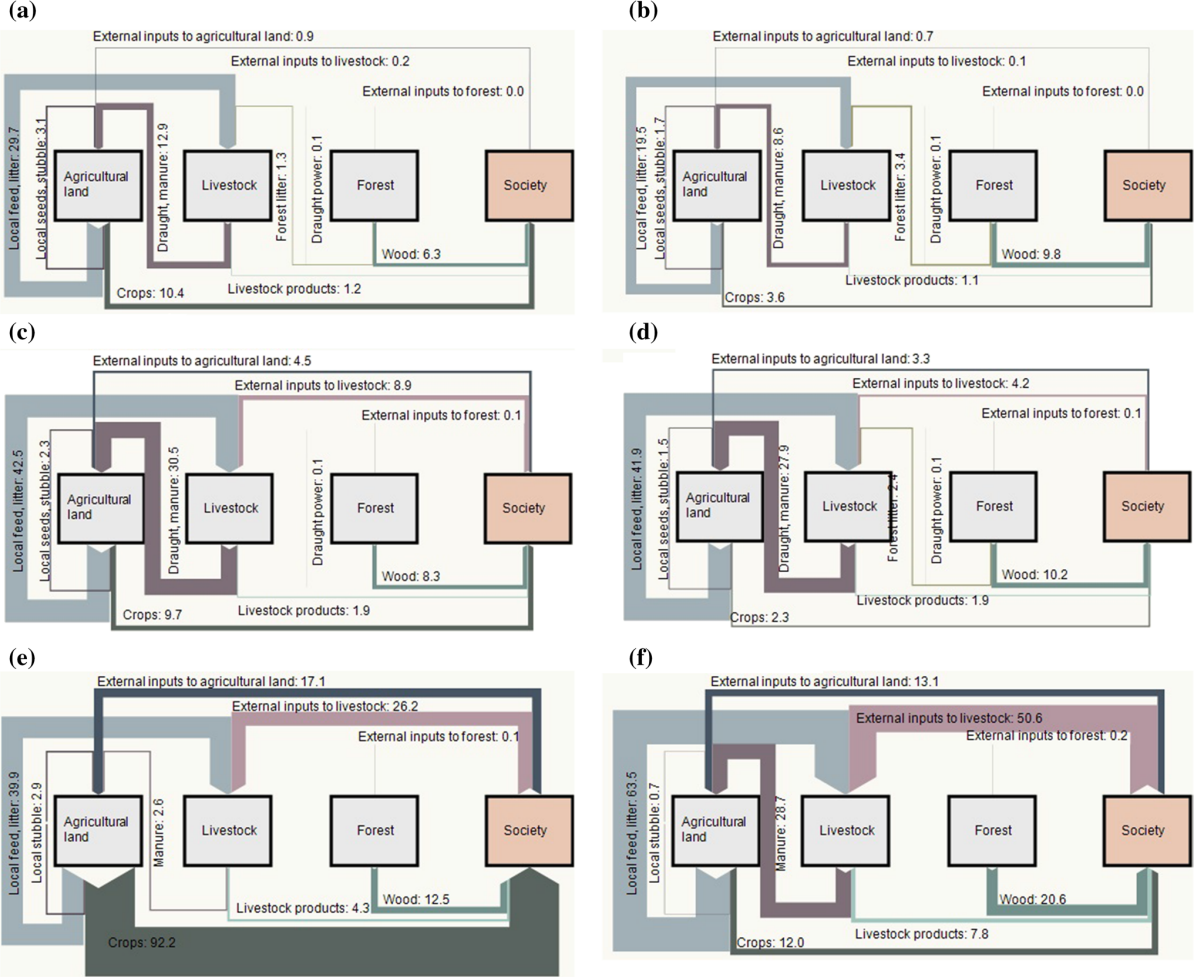
Energy flows in Sankt Florian (*left*) and Grünburg (*right*) 1830–2000, units in GJ/ha/yr; flowcharts of all years, including 1864 and 1960, are presented in the supplementary information (SI Figs. 2 and 3)

Opening the black box of the agroecosystems, we discern the origin and destination of the agroecosystem’s internal energy flows, and include flows between the compartments, i.e., flows from livestock to forest and agricultural land and vice versa. Figure 4 displays these energy flows in Sankt Florian and Grünburg for the years 1830, 1950, and 2000 (Figures for all years, including 1864 and 1960 are shown in SI Figs. 2 and 3).

In 1830, the two regions were characterized by similar energetic profiles, with little societal energy inputs and a high degree of energy flow integration between compartments. Energy was transferred from every compartment to the other. Societal activities focused on harnessing local agroecosystem energy flows between these compartments, instead of inserting much external energy. Final produce was a small flow compared to some internal agroecosystem energy transfers, but large compared to external inputs. The most pronounced difference between the regions in this period was the higher importance of wood in Grünburg. In 1864, the integrated characteristic of agriculture prevailed (see SI, Figs. 2 and 3). In the nineteenth century, agroecosystem productivity increase was achieved by slightly raising external energy inputs to increase the energy flows among agroecosystem energy compartments. Changes in management included higher livestock numbers and livestock productivity, based on higher feed availability and stable keeping, which in turn produced more manure to allow for higher crop production.

In 1950, a new dynamic set in: External energy inputs to both livestock and agricultural land were significantly higher in both regions, while crop production was similar as in 1830. This period appears to be one of crisis: Short-term cropland conversion to grassland resulted in lower agroecosystem productivity than in the nineteenth century, while external inputs were already higher. With the introduction of agricultural and forest machinery and mineral fertilizers, the integration between agroecosystem compartments started to loosen: draught power declined, and forest litter was no longer used in Sankt Florian. Crop production had recovered in Sankt Florian by 1960, while in Grünburg, a trend of cropland abandonment, and grassland expansion with cattle management set in. Draught power was further reduced in both regions, and forest litter was no longer used in either region.

By 2000, the two regional agroecosystems had developed into two entirely different energy profiles. In Sankt Florian, crop production was now the largest of all energy flows. Societal energy inputs to agricultural land were small compared to agroecosystem productivity, although they were larger than at any previous point in time. As addressed above, the high level of agroecosystem productivity is partly explained by a redirection of crop residues to external markets. While still fed on local feed to some extent, the livestock sector, now specialized in pork and chicken production, had lost its role in integrating energy (or nutrient) flows between compartments (Krausmann 2004; Gingrich et al. 2015). In Grünburg on the other hand, the largest flow in 2000 was input into the livestock compartment, comprising a high share of agroecosystem biomass reuse, but also significant amounts of external inputs. Livestock production in terms of final products was still comparatively low, given the low energy conversion efficiency of livestock (Pelletier et al. 2011).

In two very different ways, both regions display the disintegration of local energetic loops in the course of industrialization and market integration. They show the increasing relative and absolute importance of external inputs into all compartments of the agroecosystem, with different effects depending on the specialization of a region. Sankt Florian’s specialization on intensive cropping entailed a more or less unidirectional energy throughput through the agroecosystem. Grünburg’s livestock system, with the dominance of grassland-based cattle rearing, was equally dependent on external energy input, but also depended on large amounts of biomass reused in the form of feed and litter. This allowed for a certain integration of agroecosystem energy flows to persist.

## Discussion

### An agroecosystem energy transition: two stages of intensification in a Central European context

Our study confirms the observation that agricultural industrialization resulted in increasing yields at the cost of growing external energy inputs at declining (Tello et al. 2016) or relatively stable (Fraňková and Cattaneo 2017, Guzman Casado et al. 2017) amounts of biomass reused. The availability of more than two benchmark years and the comparative approach adopted allow us to trace different stages of an agroecosystem energy transition, and offer insights on major determinants of particular energetic profiles in industrial agriculture. We identify (1) a period of organic intensification in the 19th century and (2) a period of industrial intensification with regional specialization and market integration in second half of the 20th century.

In the nineteenth century, the two agroecosystems investigated had similar energy profiles, locally integrating cropping, livestock rearing, and forestry. Also, their trajectories were comparable: With energy inputs consisting of labor, biomass reused, and locally produced kitchen waste only, the only options to increase agroecosystem productivity were to increase these flows and invest in more efficient energy transfer within the agroecosystem. After World War II, both regions relied more and more on external energy inputs and were able to increase their agroecosystem productivity, while at the same time specializing their production.

 Our approach, distinguishing external and internal energy inputs, allows to characterize the second stage of the agroecosystem energy transition as an increase in a new type of energy input (i.e., more fossil-energy-based inputs), but also in the origin and destination of agroecosystem energy flows: Instead of producing locally and integrating energy flows within the regional agroecosystem, agroecosystems use more and more external inputs, and, in the case of Sankt Florian, also export more of what was previously reused locally. The second stage of the agroecosystem energy transition, according to our results, is thus characterized not only by increasing fossil energy input, but also by a partial replacement of biomass reuse by external energy inputs, as well as a partial replacement of biotic by abiotic inputs. Both processes were enabled through increasing market integration. Differences in agroecosystem energy efficiency were, in the two case studies investigated here, mostly related to the regional specialization on either cropping or livestock rearing, rather than to different energy efficiencies within these production strategies.

 The increasing reliance on external inputs resulted in shifts of environmental burdens. Previous studies have shown that Austrian agroecosystems were relieved from some pressures in the course of the socio-ecological transition, e.g., carbon stocks in agroecosystems regrew by c.20% between 1830 and 2000 (Gingrich et al. 2007) and Human Appropriation of Net Primary Production (HANPP) declined from c. 60% of net primary production to c. 50% in the same period (Krausmann 2001). On the other hand, new local environmental problems related to industrial agroecosystem inputs such as mineral fertilizer emerged (Krausmann et al. 2012). In addition, environmental burdens were externalized from local agroecosystems, either to other world regions, e.g., when feed is imported (Guyomard et al. 2013) or to the atmosphere, as in the use of fossil fuels contributing to CO_2_ accumulation in the atmosphere (Lal 2004). Our findings demonstrate that not only biomass consumption relies on ever more integrated global trade flows, as has been demonstrated in various studies on “teleconnections” (Kastner et al. 2011; Yu et al. 2013): Also biomass production is more and more dependent on non-local resources.

### Implications for sustainable land-use intensification

The agroecosystem energy transition described in our two case studies hints at a dilemma of sustainable land-use intensification (Erb et al. 2016): Under traditional organic conditions, external energy input and related external environmental pressures are low, but land productivity remains well below the levels of industrialized agriculture. Producing the same amount of agricultural products under purely organic conditions thus requires more land than in industrialized agriculture (referred to as the “land cost of sustainability” by Guzman Casado and Gonzalez de Molina 2009). On the other hand, industrialized farming generates high yields but at higher external, as well as local, environmental costs. Remote effects of industrialized agriculture include CO_2_-emissions contributing to climate change and oceanic acidification, as well as to an acceleration of global nitrogen cycles, three of the major planetary boundaries (Steffen et al. 2015).

Based on the findings of this study, we argue that the dilemma of agricultural sustainability versus productivity may not be adequately addressed by simply “re-localizing” food production and consumption. The case of Sankt Florian shows that productivity increases in specialized industrialized agriculture exceeded the growth of industrial energy inputs. Grünburg on the other hand relied on more biomass recycling, but reached much lower productivity levels because of the importance of livestock. Strategies to increase agroecosystems’ energy efficiency by solely reducing external energy inputs may thus compromise agroecosystem productivity. In our view, a certain degree of sustainable regional specialization in agricultural production seems to be more promising, allowing optimal use of local production potentials, and relying on some external inputs. Smart reductions of industrial inputs (mineral fertilizers, agricultural machinery), and shift to locally adapted crops and cropping techniques in order to achieve solid yields, are in our view potential steps in this direction. Which level of regional specialization optimizes local productivity at limited inputs, however, remains a topic for future research.

## Electronic supplementary material

Below is the link to the electronic supplementary material.

Supplementary material 1 (PDF 625 kb)

## Footnotes

1 Cadastral maps and aerial photographs of Upper Austria can be viewed at https://doris.ooe.gv.at/viewer/(S(1m0fpb2pe2esljtctcfdabvc))/init.aspx?ks=alk&karte=urmappe. Click “Orthofoto Speed” or “Urmappe Speed” to switch between the two image types.

## References

[CR1] Aguilera E, Guzmán G, Infante-Amate J, Soto D, Garcia-Ruiz R, Herrera A, Villa I, Torremocha E, Carranza G, Gonzalez de Molina M (2015) Embodied energy in agricultural inputs. Incorporating a historical perspective, Sociedad Española de Agricultura Ecológica (SEAE)

[CR59] AT-OOeLA 1830, Franziszäischer Kataster, Boxes 46, 172, 179, 197, 244, 562, 580, 652, 678, 752, 775, 826, 854, 871, 901, 997, 1030, 1068, 1112, 1120, 1143, 1191

[CR2] BMLF (1999) Richtlinien für sachgerechte Düngung. Anleitung zur Interpretation von Bodenuntersuchungsergebnissen in der Landwirtschaft. Wien

[CR3] BMLFUW (2006) Richtlinien für sachgerechte Düngung. Anleitung zur Interpretation von Bodenuntersuchungsergebnissen in der Landwirtschaft. Wien

[CR4] Boserup E (1965). The conditions of agricultural growth.

[CR5] Darge E (2001) Energieflüsse im österreichischen Landwirtschaftssektor 1950–1995, Eine humanökologische Untersuchung. Diplomarbeit an der Universität Oldenburg, Oldenburg

[CR6] Dissemond H, Zaussinger A (1995). Stroh - ein nachwachsender Rohstoff für die energetische Nutzung. Bodenkult.

[CR7] Erb KH, Lauk C, Kastner T, Mayer A, Theurl M, Haberl H (2016). Exploring the biophysical option space for feeding the world without deforestation. Nat Commun.

[CR8] Festersen J (1990). Zur Rolle der Tierproduktion und ihres Einflusses auf den Stoff- und Energiefluß im Agroökosystem. Biol Rundsch.

[CR9] Fischer-Kowalski M, Haberl H (2007). Socioecological transitions and global change: trajectories of social metabolism and land use.

[CR10] Fischer-Kowalski M, Krausmann F, Giljum S, Lutter S, Mayer A, Bringezu S, Moriguchi Y, Schütz H, Schandl H, Weisz H (2011). Methodology and indicators of economy-wide material flow accounting. J Ind Ecol.

[CR11] Fluck RC, Fluck RC (1992). Energy analysis in agricultural systems. Energy in farm production.

[CR12] Fraňková E, Cattaneo C (2017). Organic farming in the past and today: sociometabolic perspective on a Central European case study. Reg Environ Chang.

[CR13] Galan ES, Padró R, Marco I, Tello E, Cunfer G, Guzman G, Gonzalez de Molina M, Krausmann F, Gingrich S, Sacristan V, Moreno D (2016). Widening the analysis of Energy Return On Investment (EROI) in agro-ecosystems: socio-ecological transitions to industrialized farm systems (the Vallès County, Catalonia, c.1860 and 1999). Ecol Mod.

[CR14] Gingrich S, Erb K-H, Krausmann F, Gaube C, Haberl H (2007). Long-term dynamics of terrestrial carbon stocks in Austria: a comprehensive assessment of the time period from 1830 to 2000. Reg Environ Chang.

[CR15] Gingrich S, Schmid M, Gradwohl M, Krausmann F, Singh SJ, Haberl H, Chertow M, Mirtl M, Schmid M (2013). How material and energy flows change socio-natural arrangements: the transformation of agriculture in the Eisenwurzen region, 1860–2000. Long term socio-ecological research. Studies in society—nature interactions across spatial and temporal scales.

[CR16] Gingrich S, Haidvogl G, Krausmann F, Preis S, Garcia-Ruiz R (2015). Providing food while sustaining soil fertility in two pre-industrial Alpine agroecosystems. Hum Ecol.

[CR17] Gonzalez de Molina M, Toledo VM (2014). The social metabolism. A socio-ecological theory of historical change.

[CR18] Granda S, Furter R (2006). Kataster als Quelle für die Wirtschaftsgeschichte. Cultures Alpines = Alpine Kulturen.

[CR19] Guyomard H, Manceron S, Peyraud J-L (2013). Trade in feed grains, animals, and animal products: current trends, future prospects, and main issues. Anim Front.

[CR20] Guzman Casado GI, Gonzalez de Molina M (2009). Preindustrial agriculture versus organic agriculture: the land cost of sustainability. Land Use Policy.

[CR21] Guzman Casado GI, Gonzalez de Molina M, Soto Fernandez D, Infante Amate J, Aguilera E (2017). Spanish Agriculture from 1900 to 2008: a long-term perspective on agroecosystem energy from an agro-ecological approach. Reg Environ Chang.

[CR22] Haberl H (1995) Menschliche Eingriffe in den natürlichen Energiefluß von Ökosystemen: Sozio-ökonomische Aneignung von Nettoprimärproduktion in den Bezirken Österreichs. Social Ecology Working Paper 43, Vienna

[CR23] Haberl H, Winiwarter V, Andersson K, Ayres RU, Boone C, Castillo A, Cunfer G, Fischer-Kowalski M, Freudenburg WR, Furman E, Kaufmann R, Krausmann F, Langthaler E, Lotze-Campen H, Mirtl M, Redman CL, Reenberg A, Wardell A, Warr B, Zechmeister H (2006) From LTER to LTSER: conceptualizing the socioeconomic dimension of long-term socioecological research. Ecol Soc 11(2):13 [online]. http://www.ecologyandsociety.org/vol11/iss2/art13/

[CR24] Hall CAS, Balogh S, Murphy DJR (2009). What is the minimum EROI that a sustainable society must have?. Energies.

[CR25] Hamilton A, Balogh S, Maxwell A, Hall C (2013). Efficiency of edible agriculture in Canada and the U.S. over the past three and four decades. Energies.

[CR26] Hitschmann HH (1891) Vademecum für den Landwirth. M. Perles

[CR27] Hoffman A (1974). Bauernland Oberösterreich: entwicklungsgeschichte seiner Land- und Forstwirtschaft.

[CR28] Kastner T, Kastner M, Nonhebel S (2011). Tracing distant environmental impacts of agricultural products from a consumer perspective. Ecol Econ.

[CR29] Kirner L, Eder M, Schneeberger W (2002). Strukturelle Merkmale der Biobetriebe 2000 in Österreich—Vergleich zu den konventionellen Betrieben im Invekos und der Agrarstrukturerhebung. Ländlicher Raum.

[CR30] Klein Goldewijk K, Beusen A, Van Drecht G, De Vos M (2011). The HYDE 3.1 spatially explicit database of human-induced global land-use change over the past 12,000 years: HYDE 3.1 Holocene land use. Glob Ecol Biogeogr.

[CR31] Krausmann F (2001). Land use and industrial modernization: an empirical analysis of human influence on the functioning of ecosystems in Austria 1830–1995. Land Use Policy.

[CR32] Krausmann F (2004). Milk, manure, and muscle power. Livestock and the transformation of preindustrial agriculture in Central Europe. Hum Ecol.

[CR33] Krausmann F (2008) Land use and socio-economic metabolism in pre-industrial agricultural systems: four nineteenth-century Austrian villages in comparison. Social Ecology Working paper 72, Vienna

[CR34] Krausmann F, Gingrich S, Eisenmenger N, Erb KH, Haberl H, Fischer-Kowalski M (2009). Growth in global materials use, GDP and population during the 20th century. Ecol Econ.

[CR35] Krausmann F, Gingrich S, Haberl H, Erb KH, Musel A, Kastner T, Kohlheb N, Niedertscheider M, Schwarzlmüller E (2012). Long-term trajectories of the human appropriation of net primary production: lessons from six national case studies. Ecol Econ.

[CR36] Krausmann F, Erb K-H, Gingrich S, Haberl H, Bondeau A, Gaube V, Lauk C, Plutzar C, Searchinger T (2013). Global human appropriation of net primary production doubled in the 20th century. Proc Natl Acad Sci.

[CR37] Lal R (2004). Carbon emission from farm operations. Environ Int.

[CR38] Löhr L (1952). Faustzahlen für den Landwirt.

[CR39] Löhr L (1983). Faustzahlen für den Landwirt.

[CR56] Lorenz JR von (1866) Statistik der Bodenproduction von zwei Gebietsabschnitten Oberösterreichs (Umgebung von St. Florian und von Grünburg). k.k. Ministerium für Handel und Volkswirthschaft, Wien

[CR40] Mather AS, Needle CL (1998). The forest transition: a theoretical basis. Area.

[CR41] Millennium Ecosystem Assessment (2005) Ecosystems and Human Well-being. Island Press, Washington, DC

[CR42] Österreichisches Statistisches Zentralamt (1950). Ergebnisse der landwirtschaftlichen Statistik in den Jahren 1946-1949.

[CR43] Österreichisches Statistisches Zentralamt (1952). Ergebnisse der landwirtschaftlichen Statistik im Jahre 1951.

[CR44] Österreichisches Statistisches Zentralamt (1952). Ergebnisse der Volkszählung vom 1. Juni 1951 nach Gemeinden.

[CR45] Ozkan B, Akcaoz H, Fert C (2004). Energy input–output analysis in Turkish agriculture. Renew Energy.

[CR46] Pelletier N, Audsley E, Brodt S, Garnett T, Henriksson P, Kendall A, Kramer KJ, Murphy D, Nemecek T, Troell M (2011). Energy intensity of agriculture and food systems. Annu Rev Environ Resour.

[CR47] Ringhofer L, Singh SJ, Fischer-Kowalski M, Fischer-Kowalski M, Reenberg A, Schaffartzik A, Mayer A (2014). Beyond Boserup: the role of working time in agricultural development. Ester boserup’s legacy on sustainability.

[CR48] Ruhr-Stickstoff-Aktiengesellschaft (1957) Faustzahlen für die Landwirtschaft. Bochum

[CR49] Sandgruber R (1979). Der Franziszeische Kataster und die dazugehörigen Steuerschätzungsoperate als wirtschafts-und sozialhistorische Quellen. Mitteilungen aus dem niederösterreichischen Landesarch.

[CR50] Singh SJ, Haberl H, Chertow MR, Mirtl M, Schmid M (2013). Long term socio-ecological research: studies in society-nature interactions across spatial and temporal scales.

[CR51] Smil V (2000). Feeding the world. A challenge for the 21st Century.

[CR52] Steffen W, Richardson K, Rockstrom J, Cornell SE, Fetzer I, Bennett EM, Biggs R, Carpenter SR, de Vries W, de Wit CA, Folke C, Gerten D, Heinke J, Mace GM, Persson LM, Ramanathan V, Reyers B, Sorlin S (2015). Planetary boundaries: guiding human development on a changing planet. Science.

[CR53] Steinhart SS, Steinhart CE (1974). Energy use in the US food system. Science.

[CR54] Tello E, Galan ES, Cunfer G, Guzman G, Gonzalez de Molina M, Krausmann F, Gingrich S, Sacristan V, Marco I, Padró R, Moreno D (2015) A proposal for a workable analysis of Energy Return On Investment (EROI) in agroecosystems. Part I: analytical approach. Social Ecology Working Paper 156, Vienna

[CR55] Tello E, Galán E, Sacristán V, Cunfer G, Guzman G, Gonzalez de Molina M, Krausmann F, Gingrich S, Padró R, Marco I, Moreno-Delgado D (2016). Opening the black box of energy throughputs in farm systems: a decomposition analysis between the energy returns to external inputs, internal biomass reuses and total inputs consumed (the Vallès County, Catalonia, c.1860 and 1999). Ecol Econ.

[CR57] Wagner K (1990). Neuabgrenzung landwirtschaftlicher Produktionsgebiete in Österreich.

[CR58] Yu Y, Feng K, Hubacek K (2013). Tele-connecting local consumption to global land use. Glob Environ Chang.

