# Supplementary material for: Regional specialization and market integration: agroecosystem energy transitions in Upper Austria
Source: Reg Environ Change. 2017 Apr 7;18(4):937–50. doi: 10.1007/s10113-017-1145-1 (PMC6560786; doi:10.1007/s10113-017-1145-1)

Supplementary Information

Regional specialization and market integration: Agroecosystem energy transitions in Upper Austria

Simone Gingrich, Michaela Theurl, Karl-Heinz Erb, Fridolin Krausmann

Case study region

*SI – Table 1: cadastral villages in 1830**

Villages in Sankt Florian Region 1830	Number of Archival Box in Upper Austrian Provincial Archive
Ansfelden	Franziszäischer Kataster, Box 46
Rapperswinkel	Franziszäischer Kataster, Box 826
Mickstetten	Franziszäischer Kataster, Box 580
Enzing	Franziszäischer Kataster, Box 179
Rohrbach	Franziszäischer Kataster, Box 854
Gemering	Franziszäischer Kataster, Box 244
Markt Sankt Florian	Franziszäischer Kataster, Box 562
Niederfraunleiten	Franziszäischer Kataster, Box 652
Fernbach	Franziszäischer Kataster, Box 197
Samesleiten	Franziszäischer Kataster, Box 871
Taunleiten	Franziszäischer Kataster, Box 1030
Villages in Grünburg Region 1830	
Emsenhub	Franziszäischer Kataster, Box 172
Weißenbach	Franziszäischer Kataster, Box 1143
Waldneukirchen	Franziszäischer Kataster, Box 1120
Sankt Nikola	Franziszäischer Kataster, Box 901
Pieslwang	Franziszäischer Kataster, Box 775
Steinbach an der Steyr	Franziszäischer Kataster, Box 997
Obergrünburg	Franziszäischer Kataster, Box 678
Untergrünburg	Franziszäischer Kataster, Box 1068
Wagenhub	Franziszäischer Kataster, Box 1112
Zehetner	Franziszäischer Kataster, Box 1191
Pernzell	Franziszäischer Kataster, Box 752

* For Asten and Raffelstetten, two villages of Sankt Florian Region, the cadastral records are not extant.

SI – Table 2: Political communities 1950-2000

Political communities Sankt Florian Region	"Gemeindenummer"; official number of community
Ansfelden	41002
Asten	41003
Sankt Florian	41013
Political communities Grünburg Region	
Adlwang	41501
Grünburg	40902
Steinbach an der Steyr	40920
Waldneukirchen	41518

SI Figure 1: location of the study regions in the Austrian province Upper Austria. The areas refer to the 20th century village boundaries.

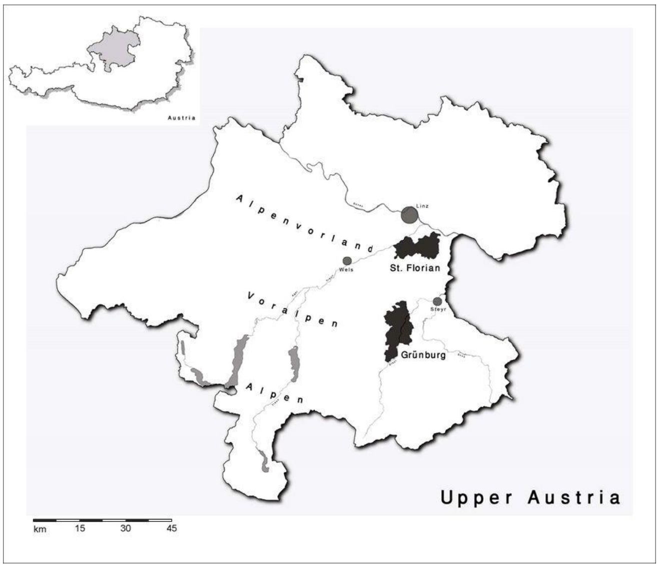

SI – Figure 2: Agroecosystem energy flows Sankt Florian (GJ/ha/yr)

Sankt Florian 1830

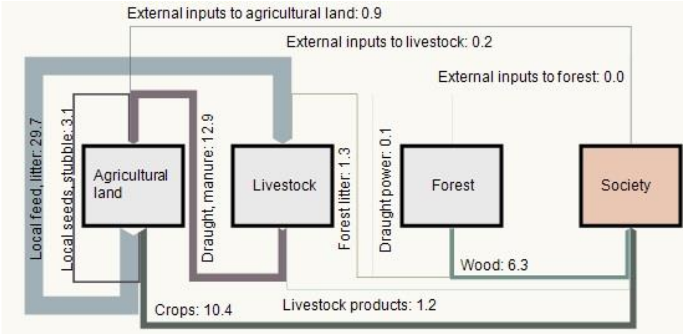

Sankt Florian 1864

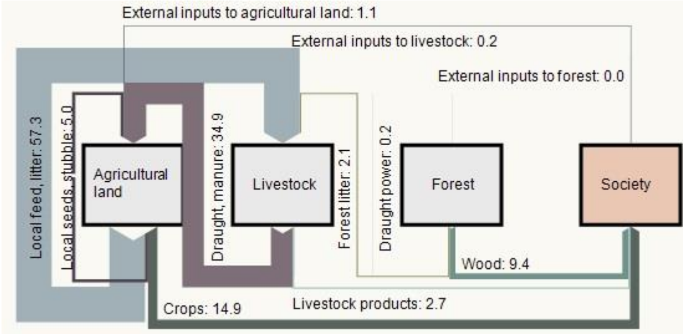

Sankt Florian 1950

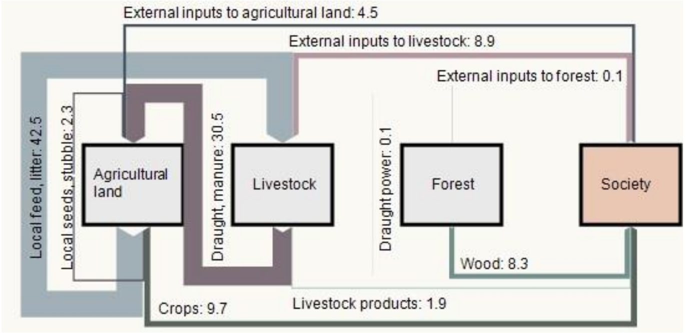

Sankt Florian 1960

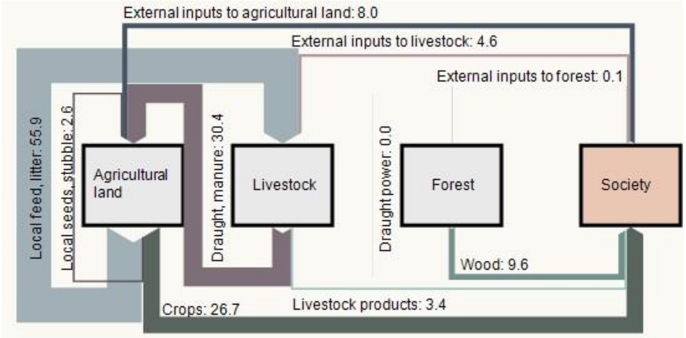

Sankt Florian 2000

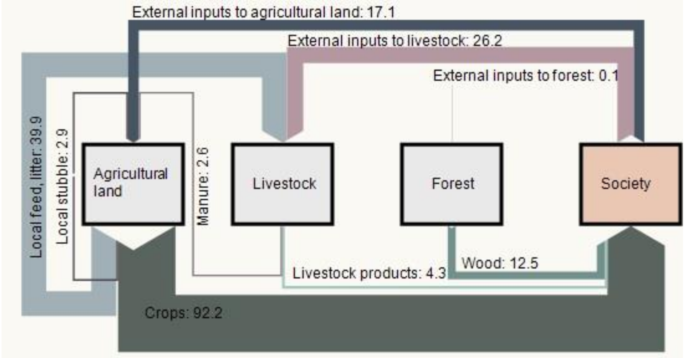

SI – Figure 3: Agroecosystem energy flows Grünburg (GJ/ha/yr)

Grünburg 1830

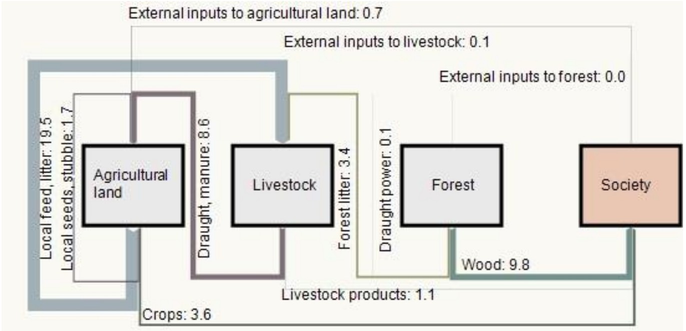

Grünburg 1864

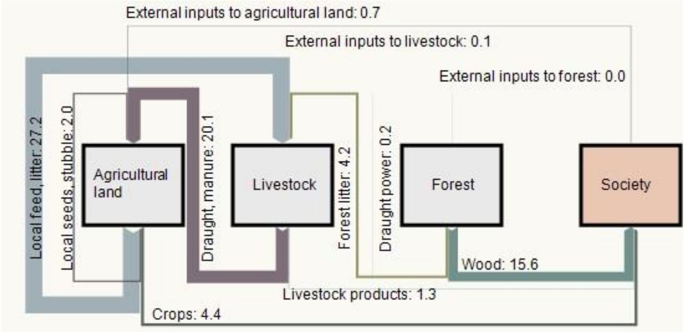

Grünburg 1950

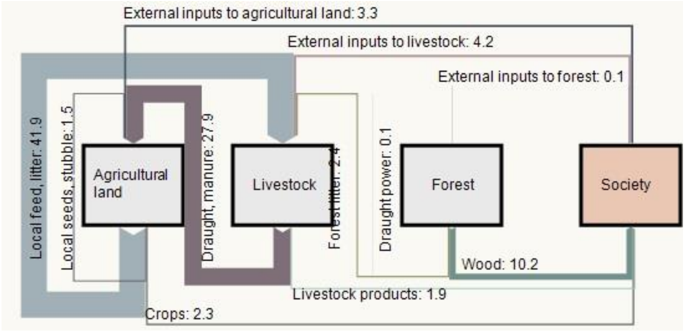

Grünburg 1960

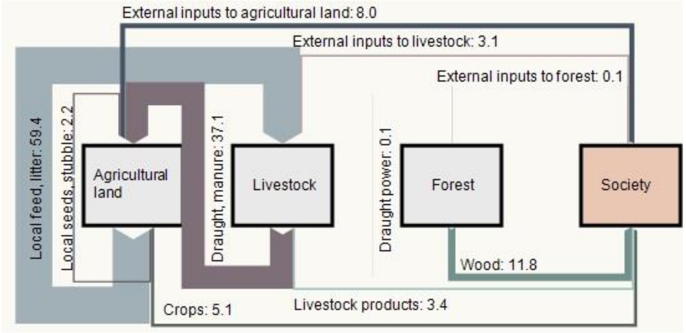

Grünburg 2000

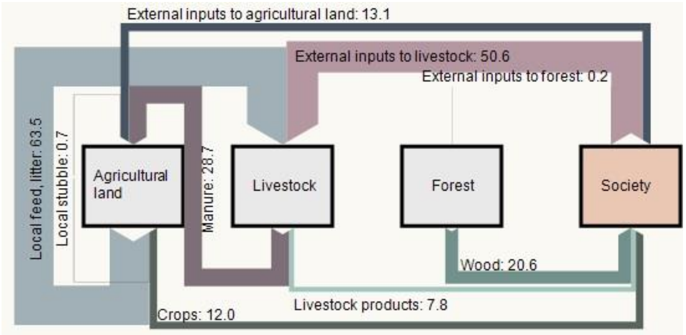

Supplement: Supplementary file 1 — Supplementary material 1 (PDF 625 kb) [file 10113_2017_1145_MOESM1_ESM.pdf]
